# Development and external validation of a metabolic and lipid-based model for predicting subsequent pregnancy in women with endometriosis

**DOI:** 10.3389/fimmu.2026.1893630

**Published:** 2026-09-01

**Authors:** Yanan Duan, Aiping Chen, Yushuang Yao

**Affiliations:** 1 Department of Gynecology, The Affiliated Hospital of Qingdao University, Qingdao, Shandong, China; 2 Qingdao Medical College, Qingdao University, Qingdao, Shandong, China

**Keywords:** endometriosis, lipid-related indicators, metabolic dysfunction, predictive model, pregnancy outcome

## Abstract

**Background:**

Endometriosis is frequently associated with impaired fertility. Metabolic abnormalities and dyslipidemia may be relevant to reproductive dysfunction among women with endometriosis; however, their associations with subsequent reproductive outcomes and their predictive value remain unclear. This study aimed to examine the associations of metabolic and lipid-related indicators with subsequently recorded delivery and to develop and validate a prediction model among women with endometriosis.

**Methods:**

We included 5,604 women of reproductive age with endometriosis from the UK Biobank and an independent cohort from the Affiliated Hospital of Qingdao University. The primary outcome in the UK Biobank was subsequently recorded delivery during follow-up, whereas the corresponding outcome in the external cohort was clinical pregnancy. Associations were evaluated using logistic regression, subgroup analysis, restricted cubic splines, and threshold-effect analysis. The UK Biobank cohort was randomly divided into training and internal validation sets at a 7:3 ratio. Predictors were selected using least absolute shrinkage and selection operator regression with 10-fold cross-validation, followed by multivariable logistic regression and nomogram construction. Model performance was evaluated in the training, internal validation, and external validation cohorts.

**Results:**

During a median follow-up of 13 years, 209 of the 5,604 women in the UK Biobank cohort had a subsequently recorded delivery. Higher body mass index, waist circumference, triglyceride levels, atherogenic index of plasma, triglyceride–glucose index, and non-high-density lipoprotein cholesterol-to-high-density lipoprotein cholesterol ratio were independently associated with a lower likelihood of subsequently recorded delivery. The final prediction model incorporated age, body mass index, Townsend deprivation index, current smoking status, educational attainment, total cholesterol, and triglyceride–glucose index. The model demonstrated areas under the receiver operating characteristic curve of 0.869 in the training set, 0.854 in the internal validation set, and 0.851 in the external validation cohort.

**Conclusions:**

Less favorable metabolic and lipid profiles were associated with a lower likelihood of subsequently recorded delivery among women with endometriosis. The model may provide complementary information for reproductive risk stratification. However, differences in outcome ascertainment between cohorts and the limited number of events warrant cautious interpretation. Further prospective, multicenter validation is required before clinical application.

## Background

Endometriosis is a cORonic, estrogen dependent inflammatory disease characterized by the presence of endometrial like tissue outside the uterine cavity (1). This disease affects about 10% of women of childbearing age and is a common cause of pelvic pain and decreased fertility. The fertility of patients may be affected by multiple factors. For example, changes in pelvic anatomy can interfere with gamete transport, local inflammation, and ovarian dysfunction may affect follicular development, while decreased endometrial receptivity and embryo implantation disorders can also reduce the chances of pregnancy (2, 3). However, the reproductive outcomes of different patients are not consistent, and it is difficult to determine their subsequent pregnancy status solely based on disease diagnosis. Therefore, it is necessary to search for clinically accessible and pregnancy related evaluation indicators. Current understanding suggests that the changes involved in endometriosis may not be limited to the pelvic region. Long term inflammation and oxidative stress can interfere with glucose and lipid metabolism; At the same time, insulin resistance and dyslipidemia may further prolong the inflammatory response tORough imbalanced secretion of adipokines, impaired endothelial function, and increased production of reactive oxygen species (4, 5). This mutual influence may extend to multiple reproductive processes, including follicular development, oocyte quality, endometrial receptivity, and embryo implantation. It is speculated that metabolic abnormalities may be a potential link between endometriosis related inflammation and differences in reproductive outcomes.

Previous studies have mostly focused on individual metabolic or lipid indicators, such as body mass index, fasting blood glucose, total cholesterol, triglycerides, low-density lipoprotein cholesterol, and high-density lipoprotein cholesterol (6). These types of indicators are easy to obtain, but each indicator usually only reflects a certain aspect of metabolic status and may not fully demonstrate the relationship between glucose metabolism, insulin resistance, and lipoprotein imbalance. In contrast, composite indices that incorporate multiple biochemical parameters into the same computational framework may be closer to the overall metabolic characteristics of patients and provide additional information in individual differentiation (7, 8). The triglyceride glucose index (TyG) is calculated from fasting triglyceride and glucose concentrations and is often used as a convenient alternative indicator for evaluating insulin resistance. The plasma atherogenic index (AIP) is expressed as the logarithm of the ratio of triglyceride to high-density lipoprotein cholesterol, which can reflect the relative balance between atherogenic lipoproteins and protective lipoproteins, and is related to small and dense low-density lipoprotein particles. The ratio of non-high-density lipoprotein cholesterol to high-density lipoprotein cholesterol (NHHR), on the other hand, synthesizes the atherogenic cholesterol burden and the potential protective effect represented by HDL. TyG, AIP, and NHHR have been used for risk assessment of cardiac metabolic diseases and some inflammatory diseases, but there is still insufficient evidence to determine whether they are associated with subsequent pregnancies in women with endometriosis (9, 10). There have been few studies comparing the above composite indices with conventional metabolic and lipid indicators in prospective cohorts, and it is not yet clear whether these indices can provide independent information for predicting pregnancy probability after baseline assessment (11–13). Current predictive research is often limited by a single study population and lacks external validation, and further investigation is needed to determine whether its results can be applied to different clinical environments.

Based on two independent cohorts, this study evaluates the relationship between routine metabolic indicators, TyG, AIP, and NHHR, and subsequent pregnancy in women with endometriosis, and further investigates possible nonlinear and threshold changes. On this basis, we established an individualized pregnancy prediction model and conducted external validation in an independent cohort. The study hypothesizes that unfavorable immune metabolic states, especially higher levels of TyG, AIP, and NHHR, may be associated with a reduced probability of subsequent pregnancies.

## Methods

### Study population and design

The UK Biobank is an ongoing population-based prospective cohort that recruited more than 500,000 participants across the United Kingdom between 2006 and 2010. For the present analysis, we identified 5,604 women of reproductive age with endometriosis at baseline. Endometriosis was identified from hospital inpatient records using International Classification of Diseases, 10th Revision (ICD-10) codes N80.0-N80.9. Participants were followed from the baseline assessment until the first documented delivery event, death, loss to follow-up, or the end of the available follow-up period, whichever occurred first.

**Figure 1:**
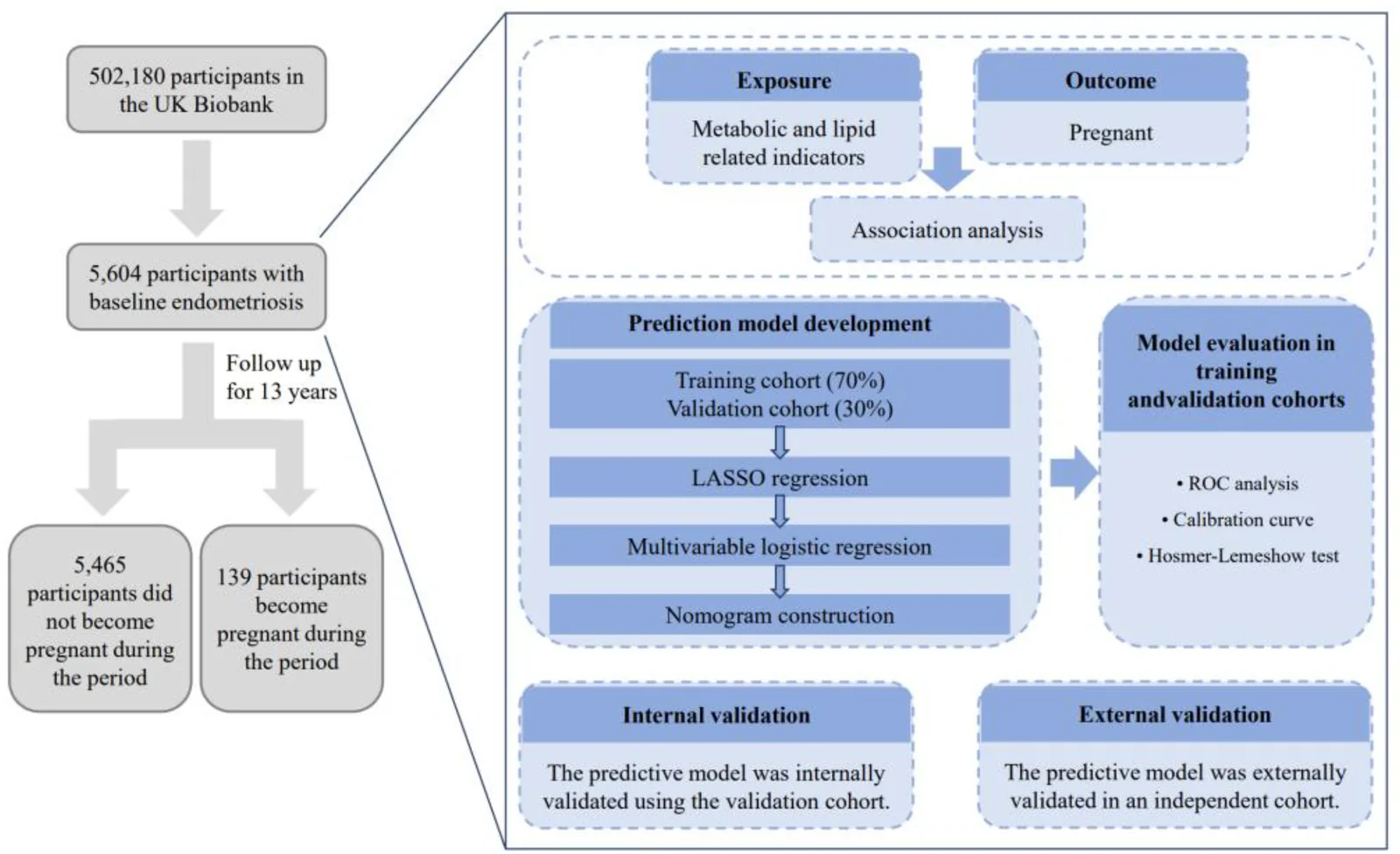
Inclusion and exclusion criteria for the study population.

The primary outcome in the UK Biobank cohort was a documented subsequent delivery, defined as the occurrence of an ICD-10 code O80-O84 after the baseline assessment. During a median follow-up of 13 years, 139 participants had a documented subsequent delivery, whereas 5,465 had no documented O80-O84 delivery record. Baseline demographic, anthropometric, metabolic, and lipid-related indicators were evaluated as exposure variables and candidate predictors. The participant-selection and analytical procedures are presented in **Figure 1**.

An independent hospital cohort comprising women with endometriosis treated at the Affiliated Hospital of Qingdao University between 2019 and 2025 was also analyzed. Because the available reproductive outcome in this cohort was clinical pregnancy rather than delivery, this cohort was not considered a strict external validation cohort. Instead, it was used for an exploratory cross-outcome transportability assessment to determine whether the UK Biobank-derived model retained predictive information for a related reproductive outcome in an independent clinical setting.

### Outcome ascertainment

Hospital inpatient diagnoses were obtained from UK Biobank Data-Field 41270 (“Diagnoses—ICD-10”), with the corresponding diagnosis dates obtained from Data-Field 41280. The primary outcome was a documented subsequent delivery, defined as the first occurrence of an ICD-10 code O80-O84 after the baseline assessment. These codes encompass spontaneous, assisted, caesarean, and multiple deliveries. Codes for pregnancy with abortive outcome (O00-O08) were excluded from the primary outcome definition. Other pregnancy-related codes outside O80-O84, including codes for hypertensive disorders, maternal or fetal conditions, complications of labor without a qualifying delivery code, and puerperal conditions, were also not regarded as evidence of the primary outcome. Participants with at least one post-baseline O80-O84 record were classified as having a documented subsequent delivery. Those without such a record were classified as having no documented subsequent delivery during follow-up. In the hospital cohort, the available outcome was clinical pregnancy. Because this outcome differed from the UK Biobank outcome, the hospital cohort was used only for a cross-outcome transportability assessment and not for strict external validation.

### Variables and definitions

The primary outcome in the UK Biobank cohort was documented subsequent delivery, defined as the first occurrence of an International Classification of Diseases, 10th Revision (ICD-10) delivery code O80–O84 after the baseline assessment. Participants were classified into the subsequent-delivery group or the no-documented-subsequent-delivery group accordingly. In the independent hospital cohort, the available outcome was clinical pregnancy, ascertained from the medical records of the Affiliated Hospital of Qingdao University. Because the reproductive outcomes differed between the two cohorts, the hospital cohort was used for an exploratory cross-outcome transportability assessment rather than strict external validation of an identical endpoint.

Baseline anthropometric and biochemical measurements were evaluated as the main exposure variables. Anthropometric variables included body mass index (BMI, kg/m^2^) and waist circumference (cm). Biochemical variables included serum creatinine (μmol/L), glucose (mmol/L), triglycerides (TG, mmol/L), total cholesterol (TC, mmol/L), low-density lipoprotein cholesterol (LDL-C, mmol/L), and high-density lipoprotein cholesterol (HDL-C, mmol/L).

Three composite metabolic and lipid-related indices were derived: the atherogenic index of plasma (AIP), triglyceride–glucose index (TyG), and non-high-density lipoprotein cholesterol-to-high-density lipoprotein cholesterol ratio (NHHR). AIP was calculated as:


$$\text{AIP}={\text{log}}_{10}[\frac{\text{TG }(\text{mmol}/\text{L})}{\text{HDL}-\text{C }(\text{mmol}/\text{L})}]$$

Because TG and HDL-C were expressed in the same molar units, their ratio was dimensionless. TyG was calculated using concentrations expressed in mg/dL:


$$\text{TyG}=\ln[\frac{\text{TG }(\text{mg}/\text{dL})\times \text{glucose }(\text{mg}/\text{dL})}{2}]$$

Before calculation, TG and glucose values recorded in mmol/L were converted to mg/dL by multiplying by 88.57 and 18.0, respectively. The natural logarithm was used for TyG. NHHR was calculated as:


$$\text{NHHR}=\frac{\text{TC}-\text{HDL}-\text{C}}{\text{HDL}-\text{C}}$$

with TC and HDL-C both expressed in mmol/L; NHHR was therefore dimensionless.

Only measurements obtained at the baseline assessment were used. This approach established a consistent temporal sequence between the exposures and the subsequent outcome, reduced the possibility that pregnancy-related physiological changes influenced the metabolic measurements, and reflected the information available at the time of prediction. Repeated measurements were not incorporated because they were not available at standardized intervals for all participants. Consequently, the estimated associations represent the relationship between baseline metabolic status and subsequent documented delivery and do not account for changes in metabolic characteristics during follow-up.

Potential confounders included age, race and ethnicity, educational attainment, smoking status, alcohol intake, and the Townsend deprivation index. Race and ethnicity were classified as White, Black, Asian, mixed, or other. Educational attainment was grouped into college or university degree, secondary education, and other qualifications. Smoking status was categorized as never, former, or current smoking, whereas alcohol intake was categorized as never, former, or current drinking.

For prediction-model development, the UK Biobank cohort was randomly divided into a training cohort and an internal validation cohort at a ratio of 7:3. Least absolute shrinkage and selection operator (LASSO) regression was performed in the training cohort to select candidate predictors. Variables retained by LASSO were subsequently entered into a multivariable logistic regression model and used for nomogram construction. The independent hospital cohort was used only for an exploratory cross-outcome transportability assessment because clinical pregnancy, rather than documented subsequent delivery, was the available outcome.

### Statistical analysis

Continuous variables are presented as mean ± standard deviation or median (interquartile range), as appropriate, and categorical variables as frequencies and percentages. Between-group comparisons were performed using Student’s *t*-test or the Mann–Whitney *U* test for continuous variables and the chi-square test or Fisher’s exact test for categorical variables.

Participants with missing values in the outcome, candidate predictors, or covariates were excluded using complete-case analysis before the cohort was divided into training and validation sets. No missing-value imputation was performed.

Associations between baseline variables and documented subsequent delivery were evaluated using logistic regression. Model I was unadjusted; Model II adjusted for age, educational attainment, and the Townsend deprivation index; Model III additionally adjusted for smoking and alcohol consumption; and Model IV further adjusted for race and ethnicity. Results are reported as odds ratios (ORs) with 95% confidence intervals (CIs). Multiplicative interaction terms were added to Model IV to assess effect modification in subgroup analyses, with corresponding $P_{\text{interaction}}$ values reported.

Nonlinear associations were assessed using restricted cubic spline logistic regression with four knots located at the 5th, 35th, 65th, and 95th percentiles of each exposure. The median exposure value was used as the reference, and all spline models were adjusted for the covariates in Model IV:


logit{P(Y=1)}=β0+RCS(X,4)+γTC

Nonlinearity was evaluated by testing the nonlinear spline terms. When significant nonlinearity was detected, two-segment logistic regression was performed, and the inflection point was selected as the value yielding the maximum model likelihood. The segmented and linear models were compared using a likelihood-ratio test. Adjusted RCS dose–response curves with 95% CIs were presented.

After complete-case selection, the cohort was randomly divided into training and internal validation sets at a 7:3 ratio. LASSO logistic regression with ten-fold cross-validation was performed in the training set. The penalty parameter corresponding to the minimum cross-validated binomial deviance was selected using cvfit$lambda.min ( $\lambda_{\text{min}}=$[insert actual value]). Multicollinearity among candidate predictors was addressed through penalized shrinkage, and only variables with nonzero LASSO coefficients at $\lambda_{\text{min}}$ were retained for multivariable logistic regression and nomogram construction. No additional VIF-based screening was performed.

Model discrimination was evaluated using ROC curves and AUCs, while calibration was assessed using calibration plots and the Hosmer–Lemeshow test. Decision-curve analysis was used to assess clinical net benefit. The hospital cohort was used for an exploratory cross-outcome transportability assessment rather than strict external validation because its outcome was clinical pregnancy rather than documented subsequent delivery. All analyses were conducted using R software. Tests were two-sided, with P<0.05 considered statistically significant.

## Result

### Participant selection and baseline characteristics

**Table 1** summarizes the baseline characteristics of the 5,604 participants. Women with a documented subsequent delivery were substantially younger than those with no documented subsequent delivery (43.28 ± 2.57 vs 51.64 ± 7.60 years, P<0.001). Given the magnitude of this difference and the strong age dependence of reproductive outcomes, age was treated as a potentially nonlinear confounder in the subsequent multivariable analyses. Compared with the no-documented-delivery group, the subsequent-delivery group had lower BMI, waist circumference, glucose, TG, TC, LDL-C, AIP, TyG, and NHHR levels (all P<0.05). Participants with a documented subsequent delivery also had a lower Townsend deprivation index and were more likely to hold a college or university degree. No statistically significant between-group differences were observed in ethnicity, smoking status, alcohol consumption, creatinine, or HDL-C.

**Table 1:** Baseline characteristics of participants.

Pregnancy status	Total	No subsequent pregnancy	Subsequent pregnancy	P-value
N	5,604	5,465	139	
Age, years	51.43 ± 7.63	51.64 ± 7.60	43.28 ± 2.57	<0.001
Ethnicity, n (%)				0.174
White	5,193 (92.86%)	5,064 (92.85%)	129 (93.48%)	
Black	131 (2.34%)	129 (2.37%)	2 (1.45%)	
Asian	134 (2.40%)	132 (2.42%)	2 (1.45%)	
Mixed	57 (1.02%)	53 (0.97%)	4 (2.90%)	
Other	77 (1.38%)	76 (1.39%)	1 (0.72%)	
Educational attainment, n (%)				<0.001
College or University degree	1,638 (29.76%)	1,571 (29.28%)	67 (48.55%)	
Secondary Education	3,341 (60.70%)	3,279 (61.11%)	62 (44.93%)	
Other professional qualifications	525 (9.54%)	516 (9.62%)	9 (6.52%)	
Smoking status, n (%)				0.565
Never	3,435 (61.61%)	3,356 (61.73%)	79 (57.25%)	
Previous	1,560 (27.98%)	1,517 (27.90%)	43 (31.16%)	
Current	580 (10.40%)	564 (10.37%)	16 (11.59%)	
Alcohol status, n (%)				0.362
Never	321 (5.75%)	316 (5.80%)	5 (3.62%)	
Previous	232 (4.15%)	224 (4.11%)	8 (5.80%)	
Current	5,032 (90.10%)	4,907 (90.09%)	125 (90.58%)	
Townsend deprivation index	−1.04 ± 3.09	−1.03 ± 3.10	−1.71 ± 2.56	0.002
Body mass index, kg/m^2^	27.80 ± 5.52	27.86 ± 5.53	25.79 ± 4.52	<0.001
Waist circumference, cm	85.86 ± 13.13	85.97 ± 13.14	81.52 ± 11.77	<0.001
Creatinine, μmol/L	63.75 ± 13.04	63.78 ± 13.13	62.56 ± 8.35	0.112
Fasting glucose, mmol/L	5.00 ± 1.00	5.00 ± 1.01	4.82 ± 0.65	0.004
TG, mmol/L	1.60 ± 0.92	1.60 ± 0.92	1.30 ± 0.75	<0.001
TC, mmol/L	5.75 ± 1.11	5.76 ± 1.11	5.27 ± 1.06	<0.001
LDL-C, mmol/L	3.57 ± 0.86	3.58 ± 0.86	3.23 ± 0.83	<0.001
HDL-C, mmol/L	1.52 ± 0.36	1.52 ± 0.36	1.50 ± 0.33	0.402
AIP	−0.02 ± 0.28	−0.02 ± 0.28	−0.11 ± 0.27	<0.001
TyG	1.24 ± 0.56	1.25 ± 0.56	1.00 ± 0.52	<0.001
NHHR	2.96 ± 1.08	2.97 ± 1.08	2.60 ± 0.96	<0.001

Univariable logistic regression results are presented in **Supplementary Table 1**. Older age was strongly associated with lower odds of documented subsequent delivery (OR 0.74; 95% CI 0.70–0.78, P<0.0001). Higher BMI, waist circumference, glucose, TG, TC, LDL-C, AIP, TyG, and NHHR were also associated with lower odds of documented subsequent delivery. These univariable associations should not be interpreted as independent effects because of the substantial differences in age and other baseline characteristics between the groups. The independent associations of the metabolic indicators were therefore evaluated in multivariable models incorporating age as a restricted cubic spline. After age was modeled using a restricted cubic spline, evidence of a nonlinear association with subsequent pregnancy was observed only for the triglyceride–glucose index (TyG, *P* = 0.041). No statistically significant nonlinear associations were identified for the other metabolic indicators (all *P* > 0.05) (**Supplementary Figure 1**).

### Associations between metabolic and lipid-related indicators and associations of metabolic and lipid-related indicators with subsequent pregnancy

**Figure 2:**
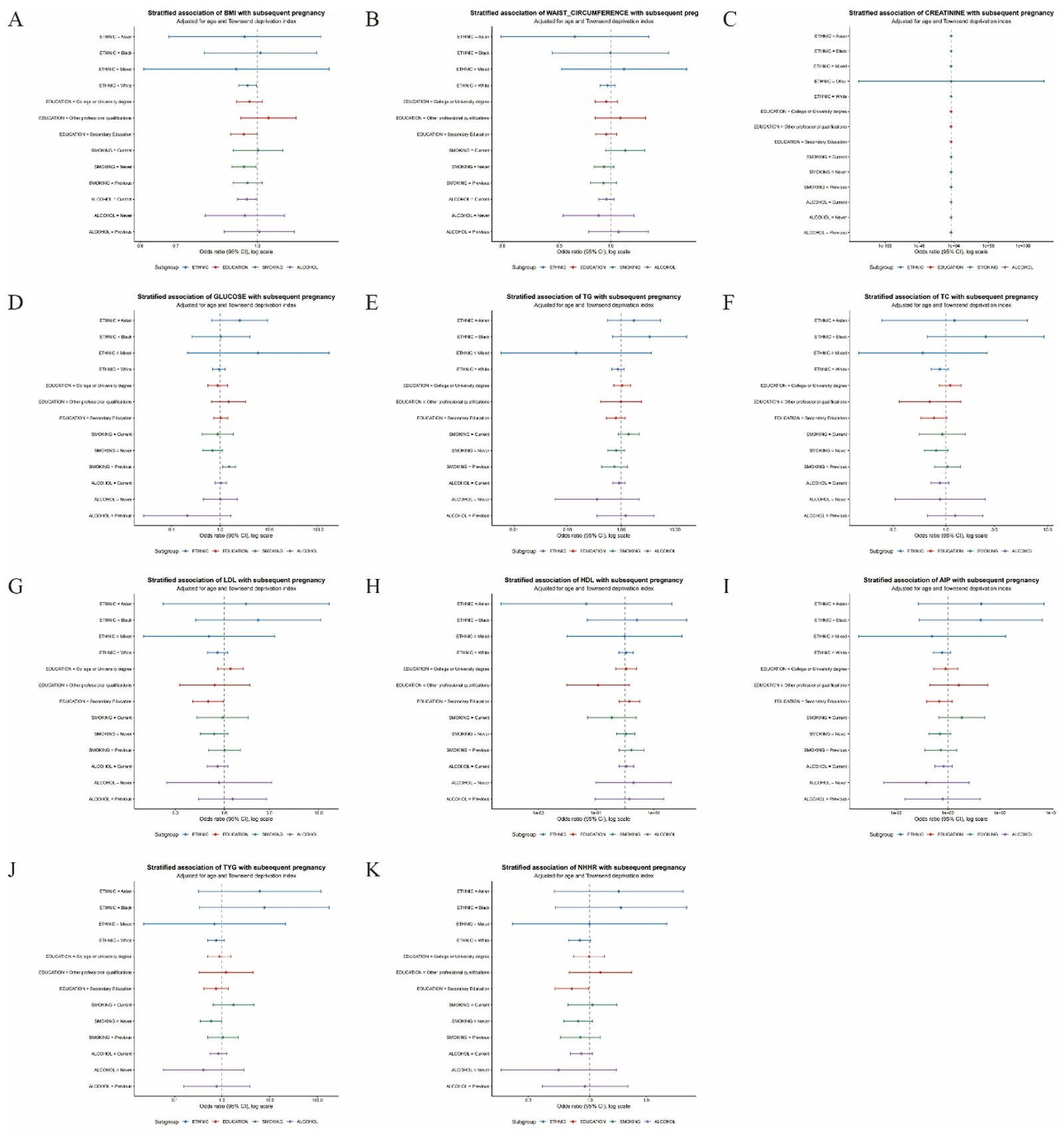
Subgroup analysis of pregnancy status in women of childbearing age with endometriosis. **(A)** BMI subgroup analysis. **(B)** Waist circumference subgroup analysis. **(C)** Creatinine subgroup analysis. **(D)** Fasting glucose subgroup analysis. **(E)** TG subgroup analysis. **(F)** TC subgroup analysis. **(G)** LDL subgroup analysis. **(H)** HDL subgroup analysis. **(I)** AIP subgroup analysis. **(J)** TyG subgroup analysis. **(K)** NHHR subgroup analysis.

The results of the logistic regression analyses are presented in **Table 2**. In the unadjusted model (Model I), higher BMI, waist circumference, fasting glucose, TG, TC, LDL-C, AIP, TyG, and NHHR were associated with lower odds of subsequent pregnancy. After adjustment for age, educational attainment, and the Townsend deprivation index in Model II, these associations were substantially attenuated. In the fully adjusted model (Model IV), only BMI remained significantly associated with subsequent pregnancy. Each 1-kg/m^2^ increase in BMI was associated with a 4% reduction in the odds of subsequent pregnancy (OR = 0.96, 95% CI: 0.92–1.00, P = 0.0365). Waist circumference, creatinine, fasting glucose, TG, TC, LDL-C, HDL-C, AIP, TyG, and NHHR were not significantly associated with subsequent pregnancy after full adjustment (all P > 0.05). These findings suggest that most associations identified in the unadjusted analyses were attenuated after accounting for potential confounding factors. The subgroup analyses are presented in **Figure 2**. The estimated associations were generally similar across the examined subgroups. However, several subgroup-specific estimates had wide confidence intervals and should therefore be interpreted cautiously. No definitive evidence of effect modification was identified across the prespecified subgroups.

**Table 2:** Multivariate analysis of pregnancy status in women of childbearing age with endometriosis.

Exposure	Model I OR (95% CI), P value	Model II OR (95% CI), P value	Model III OR (95% CI), P value	Model IV OR (95% CI), P value
BMI	0.92 (0.88, 0.95), <0.0001	0.96 (0.93, 1.00), 0.0458	0.92 (0.88, 0.95), <0.0001	0.96 (0.92, 1.00), 0.0365
Waist circumference	0.97 (0.96, 0.99), 0.0001	0.99 (0.98, 1.01), 0.3901	0.97 (0.96, 0.99), <0.0001	0.99 (0.98, 1.01), 0.3473
Creatinine	0.99 (0.97, 1.01), 0.2584	1.00 (0.98, 1.02), 0.6778	0.99 (0.97, 1.01), 0.2588	1.00 (0.98, 1.02), 0.7259
Fasting glucose	0.73 (0.54, 0.98), 0.0361	1.00 (0.77, 1.31), 0.9789	0.73 (0.54, 0.98), 0.0367	0.99 (0.76, 1.30), 0.9456
TG	0.59 (0.45, 0.77), 0.0002	0.93 (0.72, 1.20), 0.5765	0.59 (0.44, 0.77), 0.0002	0.93 (0.72, 1.20), 0.5706
TC	0.64 (0.54, 0.76), <0.0001	0.91 (0.75, 1.10), 0.3412	0.64 (0.54, 0.77), <0.0001	0.92 (0.76, 1.11), 0.3745
LDL	0.60 (0.48, 0.75), <0.0001	0.91 (0.72, 1.15), 0.4243	0.60 (0.48, 0.75), <0.0001	0.91 (0.72, 1.15), 0.4394
HDL	0.82 (0.48, 1.38), 0.4459	1.04 (0.59, 1.83), 0.8999	0.81 (0.48, 1.38), 0.4400	1.07 (0.60, 1.89), 0.8225
AIP	0.30 (0.15, 0.60), 0.0007	0.74 (0.35, 1.57), 0.4397	0.30 (0.15, 0.60), 0.0008	0.73 (0.34, 1.55), 0.4085
TyG	0.41 (0.28, 0.60), <0.0001	0.87 (0.59, 1.28), 0.4764	0.41 (0.28, 0.60), <0.0001	0.86 (0.58, 1.27), 0.4430
NHHR	0.71 (0.58, 0.87), 0.0009	0.88 (0.72, 1.08), 0.2290	0.71 (0.58, 0.87), 0.0009	0.88 (0.71, 1.08), 0.2183

Model I no adjusted.

Model II adjusted for age(smooth), educational attainment, townsend deprivation index(smooth) and educational attainment.

Model III adjusted for smoking status and alcohol status.

Model IV adjusted for age(smooth), educational attainment, townsend deprivation index(smooth), smoking status and alcohol status.

Restricted cubic spline analyses were subsequently performed with age also modeled using a restricted cubic spline (**Supplementary Figure 2**). Evidence of a nonlinear association with subsequent pregnancy was observed only for TyG (P = 0.041). No statistically significant nonlinear associations were identified for the other metabolic and lipid-related indicators (all P > 0.05). In the two-piecewise logistic regression analyses, no robust threshold effects were identified for BMI, waist circumference, fasting glucose, TG, TC, LDL-C, HDL-C, AIP, TyG, or NHHR because the corresponding likelihood-ratio tests were not statistically significant (**Supplementary Table 2**). Although the likelihood-ratio test for creatinine reached statistical significance (P = 0.0433), neither segment-specific association was statistically significant, and the confidence intervals were wide. This finding was therefore considered exploratory and should be interpreted cautiously.

### Development of the prediction model

Baseline characteristics of the training and internal validation cohorts are summarized in **Supplementary Table 3**. The 5,604 participants were randomly divided into a training cohort comprising 3,924 participants and an internal validation cohort comprising 1,680 participants at a ratio of 7:3. Subsequent pregnancy was recorded in 98 participants (2.5%) in the training cohort and 41 participants (2.4%) in the internal validation cohort. No statistically significant differences were observed in pregnancy status, demographic characteristics, lifestyle factors, or metabolic and lipid-related indicators between the two cohorts (all P > 0.05), supporting acceptable comparability after random allocation.

**Figure 3:**
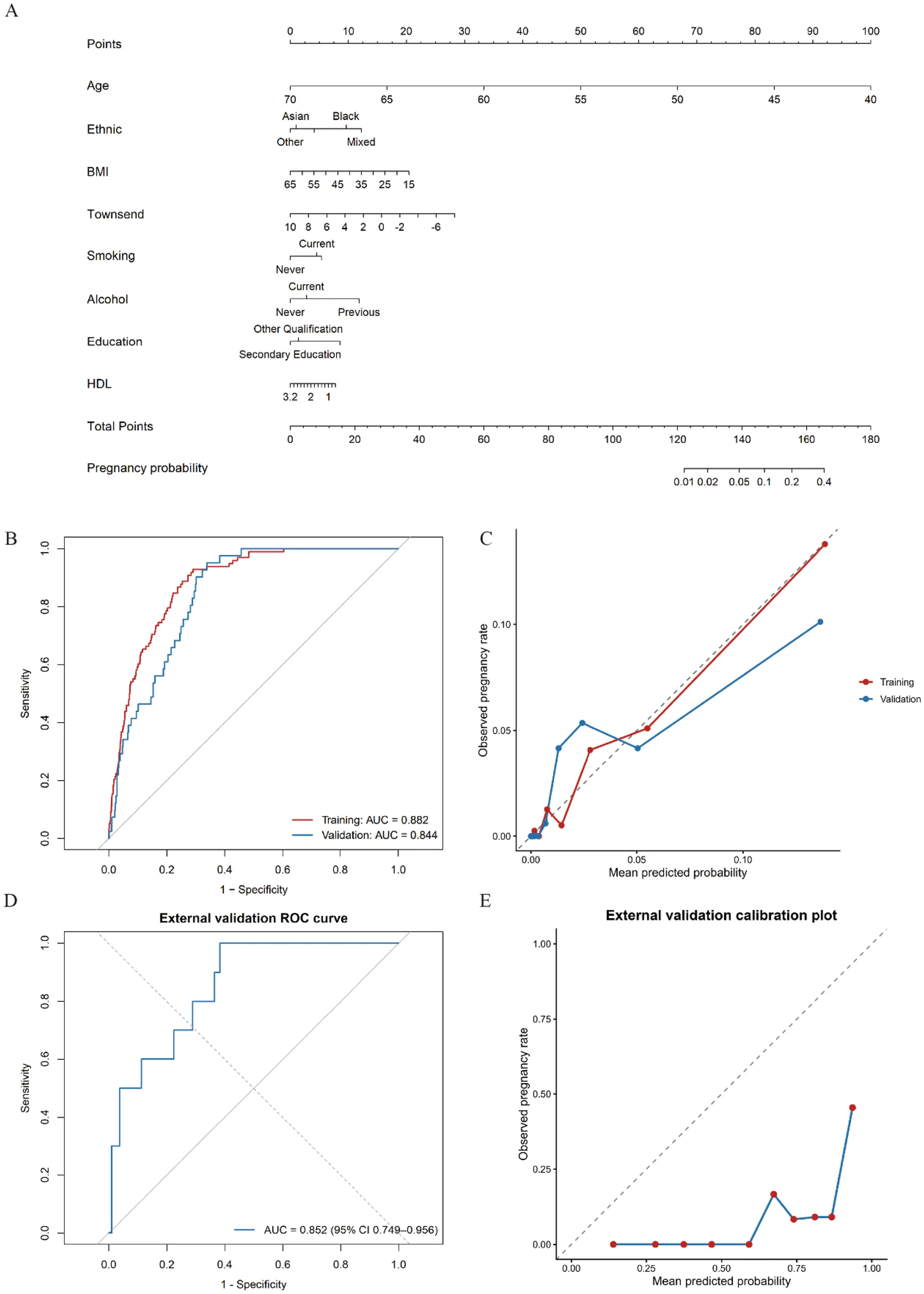
Prediction model for pregnancy status of endometriosis in women of childbearing age. **(A)** A column chart for predicting the pregnancy status of women with endometriosis of childbearing age. **(B)** ROC curves of training queue and validation queue. **(C)** Calibration curves for training and validation queues. **(D)** ROC curve of external validation queue. **(E)** Calibration curve of external validation queue. ROC curve evaluates the discriminative performance of prediction models, and calibration curve evaluates the consistency between prediction probability and observation probability.

Predictor selection was performed exclusively in the training cohort using LASSO logistic regression with ten-fold cross-validation (**Supplementary Figure 3**). A prediction model was developed using the predictors retained in the final multivariable logistic regression analysis, and a nomogram was constructed to estimate the probability of subsequent pregnancy among women of reproductive age with endometriosis (**Figure 3A**). The nomogram incorporated age, ethnicity, BMI, Townsend deprivation index, smoking status, alcohol consumption, educational attainment, and HDL-C (**Supplementary Table 4**). The total score was calculated by summing the points assigned to each predictor and was subsequently converted into an individualized predicted probability of subsequent pregnancy. The model demonstrated favorable discrimination in the training cohort, with an area under the receiver operating characteristic curve (AUC) of 0.882. In the internal validation cohort, the AUC was 0.844, indicating that the model retained acceptable discriminative performance after internal validation (**Figure 3B**). In the training dataset, calibration-in-the-large was approximately 0.000 (95% CI: −0.220 to 0.204), the calibration slope was 1.000 (95% CI: 0.838–1.179), and the Brier score was 0.0224 (95% CI: 0.0186–0.0265). In the internal validation dataset, calibration-in-the-large was 0.034 (95% CI: −0.328 to 0.342), the calibration slope was 0.745 (95% CI: 0.584–0.965), and the Brier score was 0.0231 (95% CI: 0.0171–0.0298) (**Figure 3C**; **Supplementary Table 5**). The training cohort showed relatively close agreement between the predicted and observed probabilities across most of the evaluated probability range. However, greater deviation from the ideal calibration line was observed in the internal validation cohort, particularly at higher predicted probabilities, suggesting that the model may overestimate the probability of subsequent pregnancy in some higher-risk strata. These findings indicate acceptable but imperfect internal calibration.

The external validation cohort comprised 117 women of reproductive age with endometriosis, including 10 (8.5%) who experienced subsequent pregnancy and 107 (91.5%) who did not (**Supplementary Table 6**). When applied to this independent cohort, the prediction model achieved an area under the receiver operating characteristic curve of 0.852 (95% CI: 0.749–0.956), suggesting potentially favorable discriminatory performance (**Figures 3D, E**). Nevertheless, the relatively wide confidence interval and the limited number of pregnancy events indicate considerable uncertainty in this estimate.

## Discussion

In this large-scale prospective study, we conducted a systematic analysis of the relationship between metabolic and lipid related indicators and successful pregnancy in women of childbearing age with endometriosis. Higher BMI, waist circumference, TG, AIP, TyG, and NHHR were independently associated with a lower likelihood of subsequently recorded delivery. Further analyses indicated that several indicators, particularly TyG and AIP, exhibited nonlinear associations and apparent threshold effects, suggesting that the magnitude of the associations may become more pronounced above certain levels. In addition, we developed a prediction model combining metabolic, lipid-related, and demographic variables and evaluated its performance in internal and external validation cohorts. The model showed relatively consistent discrimination, calibration, and potential net clinical benefit. Collectively, these findings suggest that adverse metabolic profiles may provide complementary information for assessing the likelihood of subsequently recorded delivery among women with endometriosis. However, the observed associations should not be interpreted as evidence of causality.

Endometriosis is not limited to gynecological diseases, but is often accompanied by systemic inflammation and metabolic abnormalities (14, 15). Previous studies have suggested that persistent inflammation, increased oxidative stress, immune dysregulation, and endocrine imbalance may be involved in endometriosis-associated reproductive dysfunction (15, 16). Previous studies have also found that obesity, insulin resistance, and dyslipidemia may reduce female fertility by affecting ovulatory function, oocyte quality, embryonic development, and endometrial receptivity (8, 17–19). In the present study, the associations of higher BMI and waist circumference with a lower likelihood of subsequently recorded delivery were broadly consistent with previous findings (20, 21). Obesity-related metabolic abnormalities may coexist with chronic inflammatory activation, altered hormone levels, and disruption of the follicular microenvironment, although these mechanisms were not directly evaluated in our study. Adipose tissue, as an important endocrine organ, can secrete various inflammatory factors, adipokines, and estrogen related mediators. These changes may further exacerbate lesion progression and reproductive dysfunction (20, 22).

In addition to conventional metabolic measurements, we evaluated several composite metabolic indices, particularly TyG, AIP, and NHHR, which capture overlapping but distinct aspects of metabolic health. TyG is commonly used as a practical surrogate indicator of insulin resistance, whereas AIP reflects the balance between triglyceride-rich and HDL-associated lipid profiles and may indirectly indicate adverse lipoprotein particle characteristics. NHHR represents the relative predominance of atherogenic cholesterol over HDL cholesterol and provides an additional measure of lipid imbalance (23–25). The research results showed that elevated levels of TyG and AIP were consistently associated with a decreased probability of successful pregnancy in multiple models and subgroup analyses. The smooth fitting curve and threshold effect analysis further suggest that these indicators may not have a simple linear relationship with pregnancy outcomes. In other words, once metabolic disorders reach a certain level, their impact on the reproductive system may become more pronounced. These findings are consistent with previous evidence linking insulin resistance and dyslipidemia to reproductive outcomes, although evidence specific to women with endometriosis remains limited. Potential explanations include mitochondrial dysfunction, oxidative stress, impaired granulosa-cell function, and reduced endometrial receptivity (26, 27). However, these mechanisms remain hypothetical in the context of the present observational study.

An additional finding of interest in the present study was the development and validation of a prediction model for successful pregnancy incorporating metabolic and lipid-related indicators. Whereas conventional reproductive prediction models predominantly emphasize hormonal profiles, ovarian reserve, and treatment-related factors, our model additionally incorporated routinely available metabolic variables. It demonstrated relatively consistent discriminatory performance across the development and validation cohorts, together with acceptable calibration and measurable net clinical benefit. Notably, findings from the independent external validation cohort further supported the reproducibility and generalizability of the model across different populations. Therefore, the model should be regarded as a potential adjunct for risk stratification rather than a substitute for conventional reproductive assessment. Larger prospective and multicenter studies are required to evaluate its incremental predictive value, recalibrate the model across populations, and determine whether its use can improve clinical decision-making.

This study has several strengths. First, it was based on a large prospective cohort with comprehensive metabolic, clinical, and lifestyle information, providing a robust basis for evaluating the associations between baseline metabolic characteristics and subsequently recorded pregnancy. Second, both conventional metabolic biomarkers and novel lipid-related composite indices were evaluated, allowing metabolic health to be assessed from complementary perspectives. Third, nonlinear, threshold-effect, and subgroup analyses were performed, and the findings were further examined in an independent validation cohort. These analyses strengthened the assessment of the consistency and reproducibility of the observed associations.

Several limitations should nevertheless be acknowledged. First, because of the observational design, the findings should be interpreted as associations rather than causal effects. Although multiple potential confounders were adjusted for, residual and unmeasured confounding cannot be excluded. Second, the UK Biobank did not provide sufficiently detailed information on several important reproductive factors, including pregnancy intention, duration of infertility, ovarian reserve, endometriosis severity, previous reproductive history, contraceptive use, frequency of sexual activity, and the use, timing, or type of assisted reproductive technology. In particular, pregnancy achieved through spontaneous conception could not be reliably distinguished from pregnancy following fertility treatment. These unmeasured factors may have influenced both metabolic characteristics and the probability of subsequent pregnancy and therefore may have affected the observed associations. Third, pregnancy outcomes were identified from ICD-10-coded healthcare records and may therefore have been affected by coding errors, incomplete ascertainment, variation in healthcare use, and the omission of pregnancies not captured by the available records. The absence of a pregnancy-related code does not necessarily indicate that pregnancy did not occur. Moreover, depending on the codes included in the outcome definition, pregnancy-related diagnoses may represent heterogeneous events rather than a uniformly successful pregnancy outcome. Such misclassification could have biased the estimated associations; although nondifferential misclassification would generally attenuate associations, its actual direction cannot be determined with certainty. Fourth, selection bias and potential collider bias cannot be excluded. Inclusion in the analytic cohort, availability of complete metabolic and reproductive information, and ascertainment of pregnancy through healthcare records may each have been influenced by both metabolic health and underlying reproductive or healthcare-seeking characteristics. Restricting the analysis to participants satisfying these conditions may therefore have induced or distorted associations between metabolic indicators and recorded pregnancy. Because several determinants of cohort inclusion and pregnancy ascertainment were unavailable, the magnitude and direction of this potential bias could not be quantified. Fifth, reverse causation and health-selection effects remain possible. Although metabolic measurements preceded the recorded pregnancy outcomes, women who subsequently became pregnant may have differed systematically in pregnancy intention, reproductive potential, healthcare-seeking behavior, underlying health status, or preconception lifestyle modification. Thus, temporal ordering alone does not establish that metabolic status affected pregnancy occurrence. Sixth, the analyses were primarily based on metabolic measurements obtained at baseline and did not capture changes in metabolic status during follow-up. Finally, although an independent validation cohort was included, its sample size and number of pregnancy events were relatively limited. Larger multicenter prospective studies incorporating clinically verified reproductive outcomes, infertility duration, ART exposure, pregnancy intention, and repeated metabolic measurements are needed to confirm these findings.

Taken together, the present findings indicate that unfavorable metabolic and lipid-related profiles may be linked to reduced likelihoods of successful pregnancy among reproductive-aged women with endometriosis. Among them, TyG and AIP showed a relatively stable association with reproductive outcomes. In addition, this study established and completed an externally validated successful pregnancy prediction model, integrating metabolic, lipid related, and demographic variables, and demonstrating stable predictive performance. These results may provide some reference for the reproductive risk assessment and subsequent personalized management of women with endometriosis.

## Data Availability

The original contributions presented in the study are included in the article/**Supplementary Material**. Further inquiries can be directed to the corresponding authors.
