# Supplementary material for: Development and external validation of a metabolic and lipid-based model for predicting subsequent pregnancy in women with endometriosis
Source: Front Immunol. 2026 Sep 1;17:1893630. doi: 10.3389/fimmu.2026.1893630 (PMC13574802; doi:10.3389/fimmu.2026.1893630)
Supplement: Supplementary file 1 [file DataSheet1.docx]

Supplementary Table 1. Univariate analysis of pregnancy status in women of childbearing age with endometriosis

|  | Pregnancy status  OR (95% CI), P value |
| --- | --- |
| Age, years | 0.74 (0.70, 0.78), <0.0001 |
| Ethnicity, n (%) |  |
| White | 1.0 |
| Black | 0.61 (0.15, 2.49), 0.4891 |
| Asian | 0.59 (0.15, 2.43), 0.4691 |
| Mixed | 2.96 (1.06, 8.31), 0.0390 |
| Other | 0.52 (0.07, 3.74), 0.5129 |
| Educational attainment, n (%) |  |
| College or University degree | 1.0 |
| Secondary Education | 0.44 (0.31, 0.63), <0.0001 |
| Other professional qualifications | 0.41 (0.20, 0.83), 0.0126 |
| Smoking status, n (%) |  |
| Never | 1.0 |
| Previous | 1.20 (0.83, 1.75), 0.3333 |
| Current | 1.21 (0.70, 2.08), 0.5019 |
| Alcohol status, n (%) |  |
| Never | 1.0 |
| Previous | 2.26 (0.73, 6.99), 0.1580 |
| Current | 1.61 (0.65, 3.96), 0.3002 |
| Townsend deprivation index | 0.92 (0.87, 0.98), 0.0102 |
| Body mass index, kg/m^2^ | 0.92 (0.88, 0.95), <0.0001 |
| Waist circumference, cm | 0.97 (0.96, 0.99), 0.0001 |
| Creatinine, μmol/L | 0.99 (0.97, 1.01), 0.2584 |
| Fasting glucose, mmol/L | 0.73 (0.54, 0.98), 0.0361 |
| TG, mmol/L | 0.59 (0.45, 0.77), 0.0002 |
| TC, mmol/L | 0.64 (0.54, 0.76), <0.0001 |
| LDL-C, mmol/L | 0.60 (0.48, 0.75), <0.0001 |
| HDL-C, mmol/L | 0.82 (0.48, 1.38), 0.4459 |
| AIP | 0.30 (0.15, 0.60), 0.0007 |
| TyG | 0.41 (0.28, 0.60), <0.0001 |
| NHHR | 0.71 (0.58, 0.87), 0.0009 |

Supplementary Table 2. Analysis of saturation threshold effect

| Exposure: | BMI | Waist circumference | Creatinine | Fasting glucose | TG | TC | LDL | HDL | AIP | TyG | NHHR |
| --- | --- | --- | --- | --- | --- | --- | --- | --- | --- | --- | --- |
| Model I |  |  |  |  |  |  |  |  |  |  |  |
| A straight line effect | 0.96 (0.92, 1.00), 0.0365 | 0.99 (0.98, 1.01), 0.3473 | 1.00 (0.98, 1.02), 0.7259 | 0.99 (0.76, 1.30), 0.9456 | 0.93 (0.72, 1.20), 0.5706 | 0.92 (0.76, 1.11), 0.3745 | 0.91 (0.72, 1.15), 0.4394 | 1.07 (0.60, 1.89), 0.8225 | 0.73 (0.34, 1.55), 0.4085 | 0.86 (0.58, 1.27), 0.4430 | 0.88 (0.71, 1.08), 0.2183 |
| Model II |  |  |  |  |  |  |  |  |  |  |  |
| Folding point (K) | 32.95 | 99.87 | 75.66 | 4.16 | 0.63 | 6.68 | 4.37 | 2.00 | 0.23 | 0.42 | 2.95 |
| <K-segment effect 1 | 0.98 (0.93, 1.03), 0.3370 | 1.00 (0.98, 1.02), 0.9022 | 1.01 (0.99, 1.04), 0.4163 | 0.77 (0.19, 3.16), 0.7150 | - | 0.84 (0.67, 1.05), 0.1164 | 0.85 (0.64, 1.12), 0.2440 | 1.51 (0.75, 3.05), 0.2521 | 0.49 (0.20, 1.23), 0.1291 | 10.10 (0.21, 482.95), 0.2413 | 0.67 (0.45, 0.99), 0.0466 |
| >K-segment effect 2 | 0.90 (0.77, 1.05), 0.1780 | 0.94 (0.87, 1.02), 0.1673 | 0.87 (0.74, 1.03), 0.1155 | 1.01 (0.76, 1.34), 0.9399 | 0.87 (0.66, 1.15), 0.3273 | 1.28 (0.83, 1.99), 0.2667 | 1.23 (0.66, 2.30), 0.5099 | 0.05 (0.00, 3.38), 0.1646 | 5.61 (0.38, 82.42), 0.2087 | 0.74 (0.47, 1.17), 0.1997 | 1.13 (0.79, 1.60), 0.5110 |
| The difference in effect between 2 and 1 | 0.92 (0.77, 1.10), 0.3731 | 0.94 (0.86, 1.03), 0.2039 | 0.86 (0.72, 1.03), 0.1077 | 1.32 (0.29, 5.92), 0.7206 | 0.00 (0.00, 9.03), 0.1236 | 1.53 (0.89, 2.66), 0.1270 | 1.46 (0.68, 3.10), 0.3289 | 0.03 (0.00, 2.96), 0.1379 | 11.43 (0.48, 274.07), 0.1329 | 0.07 (0.00, 4.13), 0.2044 | 1.68 (0.88, 3.18), 0.1137 |
| Predicted value of equation at inflection point | −5.36 (−5.96, −4.75) | −5.16 (−5.76, −4.56) | −5.12 (−5.74, −4.50) | −5.38 (−5.99, −4.77) | −5.18 (−5.75, −4.61) | −5.52 (−6.09, −4.94) | −5.46 (−6.05, −4.88) | −5.12 (−5.78, −4.47) | −5.68 (−6.35, −5.01) | −5.15 (−5.81, −4.49) | −5.61 (−6.25, −4.97) |
| Log Likelihood Ratio Tests | 0.3427 | 0.1625 | 0.0433 | 0.7266 | 0.0756 | 0.1558 | 0.3612 | 0.0812 | 0.1541 | 0.1657 | 0.1206 |

Adjusted variables: age(smooth), educational attainment, townsend deprivation index(smooth), educational attainment, smoking status and alcohol status.

Supplementary Table 3. Baseline features of the train set and validation set for the prediction model

| Group | Train set | Validation set | P-value |
| --- | --- | --- | --- |
| N | 3,924 | 1,680 |  |
| Pregnancy status, n (%) |  |  | 0.975 |
| No pregnant | 3,826 (97.5%) | 1,639 (97.6%) |  |
| Pregnant | 98 (2.5%) | 41 (2.4%) |  |
| Age, years | 51.35 ± 7.66 | 51.62 ± 7.58 | 0.212 |
| Ethnicity, n (%) |  |  | 0.525 |
| White | 3,633 (92.6%) | 1,572 (93.6%) |  |
| Black | 99 (2.5%) | 32 (1.9%) |  |
| Asian | 93 (2.4%) | 41 (2.4%) |  |
| Mixed | 41 (1.0%) | 16 (1.0%) |  |
| Other | 58 (1.5%) | 19 (1.1%) |  |
| Educational attainment, n (%) |  |  | 0.766 |
| College or University degree | 1,136 (29.0%) | 502 (29.9%) |  |
| Secondary Education | 2,417 (61.6%) | 1,024 (61.0%) |  |
| Other professional qualifications | 371 (9.5%) | 154 (9.2%) |  |
| Smoking status, n (%) |  |  | 0.593 |
| Never | 2,441 (62.2%) | 1,023 (60.9%) |  |
| Previous | 1,077 (27.4%) | 483 (28.8%) |  |
| Current | 406 (10.3%) | 174 (10.4%) |  |
| Alcohol status, n (%) |  |  | 0.492 |
| Never | 230 (5.9%) | 91 (5.4%) |  |
| Previous | 169 (4.3%) | 63 (3.8%) |  |
| Current | 3,525 (89.8%) | 1,526 (90.8%) |  |
| Townsend deprivation index | −1.04 ± 3.08 | −1.05 ± 3.09 | 0.915 |
| Body mass index, kg/m^2^ | 27.81 ± 5.57 | 27.76 ± 5.33 | 0.725 |
| Waist circumference, cm | 84.00 [76.00, 94.00] | 84.00 [76.00, 93.00] | 0.690 |
| Creatinine, μmol/L | 62.50 [57.10, 69.00] | 62.50 [57.10, 68.30] | 0.434 |
| Fasting glucose, mmol/L | 4.86 [4.62, 5.11] | 4.86 [4.60, 5.13] | 0.928 |
| TG, mmol/L | 1.36 [0.99, 1.91] | 1.36 [1.00, 1.90] | 0.660 |
| TC, mmol/L | 5.75 ± 1.08 | 5.75 ± 1.06 | 0.886 |
| LDL-C, mmol/L | 3.56 ± 0.83 | 3.56 ± 0.83 | 0.967 |
| HDL-C, mmol/L | 1.51 ± 0.33 | 1.52 ± 0.33 | 0.550 |
| AIP | −0.03 ± 0.26 | −0.02 ± 0.25 | 0.660 |
| TyG | 1.23 ± 0.51 | 1.24 ± 0.50 | 0.493 |
| NHHR | 2.93 ± 0.98 | 2.93 ± 1.00 | 0.934 |

Supplementary Table 4. LASSO regression non zero coefficient

| Variable | Coefficient |
| --- | --- |
| Age | −0.278090380 |
| Ethnic_Black | . |
| Ethnic_Asian | −0.039674612 |
| Ethnic_Mixed | 0.594630726 |
| Ethnic_Other | . |
| BMI | −0.024656662 |
| Townsend deprivation index | −0.121792271 |
| TC | . |
| Fasting glucose | . |
| LDL | . |
| TG | . |
| Waist_circumference | . |
| Creatinine | . |
| Smoking status _Previous | 0.329429819 |
| Smoking status _Current | 0.202649500 |
| Alcohol status _Previous | 0.643665055 |
| Alcohol status _Current | . |
| Education attainment _Secondary Education | −0.646353138 |
| Education attainment _Other Qualification | −0.348262557 |
| HDL | −0.025425914 |
| AIP | . |
| TyG | . |
| NHHR | . |

Supplementary Table 5. The discrimination and calibration performance of the final prediction model in the training set and internal validation set

| Dataset | N | Events | AUC (95% CI) | Calibration-in-the-large (95% CI) | Calibration slope (95% CI) | Brier score (95% CI) |
| --- | --- | --- | --- | --- | --- | --- |
| Training | 3,924 | 98 | 0.882 (0.856–0.907) | 0.000 (−0.220 to 0.204) | 1.000 (0.838–1.179) | 0.0224 (0.0186–0.0265) |
| Internal validation | 1,680 | 41 | 0.844 (0.804–0.883) | 0.034 (−0.328 to 0.342) | 0.745 (0.584–0.965) | 0.0231 (0.0171–0.0298) |

Supplementary Table 6. Baseline features of external validation set for prediction model

|  | Mean+SD / N(%) |
| --- | --- |
| Pregnancy status, n (%) |  |
| No pregnant | 107 (91.45%) |
| Pregnant | 10 (8.55%) |
| Age, years | 33.41 ± 4.22 |
| Smoking status, n (%) |  |
| Never | 116 (99.15%) |
| Previous | 1 (0.85%) |
| Alcohol status, n (%) |  |
| Never | 117 (100%) |
| Previous | 0 |
| Body mass index, kg/m^2^ | 22.21 ± 3.26 |
| Waist circumference, cm | 85.62 ± 59.23 |
| Creatinine, μmol/L | 76.28 ± 14.43 |
| Fasting glucose, mmol/L | 4.89 ± 0.39 |
| TG, mmol/L | 0.90 ± 0.63 |
| TC, mmol/L | 4.82 ± 0.89 |
| LDL-C, mmol/L | 2.61 ± 0.72 |
| HDL-C, mmol/L | 1.66 ± 0.29 |
| AIP | -0.32 ± 0.26 |
| TyG | 0.65 ± 0.52 |
| NHHR | 1.96 ± 0.61 |

Supplementary Figure 1. Restricted cubic spline analysis of the associations between metabolic indicators and subsequent pregnancy

(A) BMI. (B) Waist circumference. (C) Creatinine. (D) Fasting glucose. (E) TG. (F) TC. (G) LDL. (H) HDL. (I) AIP. (J) TyG. (K) NHHR. The red solid lines represent the adjusted odds ratios (ORs), and the blue shaded areas indicate the corresponding 95% confidence intervals. The horizontal dashed line represents an OR of 1.00, and the vertical dotted line indicates the reference value. Histograms below each curve show the distribution of the corresponding indicator. P values for the overall and nonlinear associations are displayed in each panel. Models were adjusted for age and the Townsend deprivation index as smooth terms, as well as ethnicity, educational attainment, smoking status, and alcohol consumption.

Supplementary Figure 2. Smooth Fitting Curve

(A) BMI. (B) Waist circumference. (C) Creatinine. (D) Fasting glucose. (E) TG. (F) TC. (G) LDL. (H) HDL. (I) AIP. (J) TyG. (K) NHHR. The red solid lines represent the estimated effects, and the blue dashed lines indicate the 95% confidence intervals. The distribution of each variable is shown by the rug plot along the x-axis.

Adjusted variables: age(smooth), educational attainment, townsend deprivation index(smooth), educational attainment, smoking status and alcohol status.

Supplementary Figure 2. LASSO Regression and Ten fold Cross Validation

(A) Ten-fold cross-validation plot for tuning parameter selection in the LASSO regression model. The red dots represent the mean binomial deviance values, and the gray vertical bars indicate the standard errors. The two dotted vertical lines represent the optimal values based on the minimum criteria and the 1-standard-error (1-SE) criteria. (B) LASSO coefficient profiles of candidate variables plotted against log(λ). Each colored curve represents the coefficient trajectory of an individual variable as the penalty parameter λ changes.
